# Determinants encoding fimbriae type 1 in fecal *Escherichia coli* are associated with increased frequency of bacteriocinogeny

**DOI:** 10.1186/s12866-015-0530-5

**Published:** 2015-10-06

**Authors:** Barbora Štaudová, Lenka Micenková, Juraj Bosák, Kristýna Hrazdilová, Eva Slaninková, Martin Vrba, Alena Ševčíková, Darina Kohoutová, Vladana Woznicová, Jan Bureš, David Šmajs

**Affiliations:** Department of Biology, Faculty of Medicine, Masaryk University, Kamenice 5, Building A6, 625 00 Brno, Czech Republic; Department of Infectious Diseases and Microbiology, Faculty of Veterinary Medicine, University of Veterinary and Pharmaceutical Sciences Brno, Palackého tř. 1/3, 612 42 Brno, Czech Republic; CEITEC - Central European Institute of Technology, University of Veterinary and Pharmaceutical Sciences Brno, Palackého tř. 1/3, 612 42 Brno, Czech Republic; Department of Clinical Microbiology, Faculty Hospital Brno, Jihlavská 20, 625 00 Brno, Czech Republic; 2nd Department of Internal Medicine - Gastroenterology, Charles University in Praha, Faculty of Medicine at Hradec Kralové, University Teaching Hospital, Sokolská 581, Hradec Kralové, 500 05 Czech Republic; Department of Microbiology, Faculty of Medicine, Masaryk University and St. Anne’s University Hospital, Pekařská 53, 656 91 Brno, Czech Republic

**Keywords:** *Escherichia coli*, Colicin, Microcin, Bacteriocin, Type 1 fimbriae, Phylogenetic group

## Abstract

**Background:**

To screen whether *E. coli* strains encoding type 1 fimbriae, isolated from fecal microflora, produce bacteriocins more often relative to *fimA*-negative *E. coli* strains of similar origin.

**Methods:**

PCR assays were used to detect presence of genes encoding 30 bacteriocin determinants (23 colicin- and 7 microcin-encoding genes) and 18 virulence determinants in 579 *E. coli* strains of human and animal origin isolated from hospitals and animal facilities in the Czech and Slovak Republic. *E. coli* strains were also classified into phylogroups (A, B1, B2 and D).

**Results:**

*fimA*-negative *E. coli* strains (defined as those possessing none of the 18 tested virulence determinants) were compared to *fimA*-positive *E. coli* strains (possessing *fimA* as the only detected virulence determinant). Strains with identified bacteriocin genes were more commonly found among *fimA*-positive *E. coli* strains (35.6 %) compared to *fimA*-negative *E. coli* strains (21.9 %, *p* < 0.01) and this was true for both colicin and microcin determinants (*p* = 0.02 and *p* < 0.01, respectively). In addition, an increased number of strains encoding colicin E1 were found among *fimA*-positive *E. coli* strains (*p* < 0.01).

**Conclusions:**

*fimA*-positive *E. coli* strains produced bacteriocins (colicins and microcins) more often compared to *fimA*-negative strains of similar origin. Since type 1 fimbriae of *E. coli* have been shown to mediate adhesion to epithelial host cells and help colonize the intestines, bacteriocin synthesis appears to be an additional feature of colonizing *E. coli* strains.

**Electronic supplementary material:**

The online version of this article (doi:10.1186/s12866-015-0530-5) contains supplementary material, which is available to authorized users.

## Background

*Escherichia coli* (*E. coli*) is a common, variable, aerobic bacterial species that inhabits the gut of vertebrates [1]. Strains of *E. coli* differ in a number of important characteristics including genome size [2], gene content and virulence [3]. *E. coli* strains are classified into four phylogroups; *E. coli* strains of phylogroups A and B1 contain smaller genomes and are frequently non-pathogenic, while strains of phylogroups B2 and D encode more genes and are more often pathogenic [2, 4–6].

**Fig. 1:**
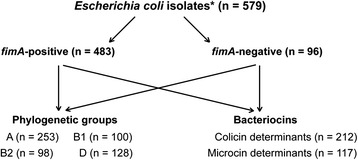
Flow chart of the *E. coli* isolates investigated in this study. *Fecal *E. coli* strains used in this study (*n* = 579) either tested negative for all 18 virulence determinants (*pCVD432, α-hly*, *afaI*, *aer*, *cnf1*, *sfa*, *pap*, *ial*, *lt*, *st*, *bfpA, eaeA, ipaH, iucC, fimA, stx1*, *stx2* and *ehly*) and were, therefore, considered *fimA*-negative, or the strains were *fimA*-positive, while still testing negative for all other virulence determinants

Approximately 80 % of all *E. coli* strains of fecal origin are able to produce type 1 fimbriae, which are encoded by the chromosomal *fim* operon [7]. This operon consists of *fimAICDFGH* genes [8], where *fimA* encodes the major fimbrial subunit (FimA). FimA is arranged in a helical manner along with minor components, one of which includes FimH (an adhesin that mediates attachment of type 1 fimbriae to mannose-containing receptors) [9]. *E. coli* type 1 fimbriae have been shown to mediate adhesion to a number of host cell types (e.g. epithelial and endothelial cells), thus potentially increasing virulence of *fimA*-positive *E. coli* strains [10].

*E. coli* strains are able to synthesize two types of bacteriocins - colicins and microcins. While microcins are low molecular weight oligopeptides, colicins are proteins with molecular weights between 30–70 kDa. Colicins and microcins differ in a number of additional parameters including operon organization, regulation of gene expression, export from producer cells, presence of post-translational modifications, antimicrobial activity, etc. [11–13]. However, this classification is not strict, since colicin Js [14, 15] is known to share features of both bacteriocin types. Several bacteriocins (i.e. colicins E1, Ia and S4, and microcins B17, E492, H47, I47, M and V) have been found to be associated with virulence factors (i.e. with *aer*, *cnf1*, *fyuA*, *hlyA*, *iroCDN*, *iucC*, *papCG*, *sfa*, *tcpC* and *usp* determinants) in *E. coli* strains [16–20].

Previous studies have found associations between several genes encoding bacteriocin types and several virulence determinants, however, no association between type 1 fimbriae and bacteriocin genes has been identified [16–20]. The role of fimbriae type 1 in the *E. coli* virulence is not clear [21, 22]. This situation is likely a result of very frequent presence of type 1 fimbriae among *E. coli* strains (over 80 %) and the fact that in most *E. coli* strains the type 1 fimbriae are combined with other virulence determinants. Therefore we collected a set of fecal *E. coli* strains encoding type 1 fimbriae as the only detected virulence determinant (out of 18 tested) and a set of *E. coli* strains with no virulence determinants (out of 18 tested).

In this communication, we studied prevalence of bacteriocin production and prevalence of bacteriocin types in both sets of *E. coli* strains to assess association of fimbriae type I encoding genes and bacteriocin determinants in *E. coli* strains. In addition, we also tested association between type 1 fimbriae determinants and other factors including biochemical profiles and *E. coli* phylogroups.

## Results

### Characteristics of *E. coli* strains

Fecal *E. coli* strains used in this study (*n* = 579) (Fig. 1) either tested negative for all 18 virulence determinants (*pCVD432, α-hly*, *afaI*, *aer*, *cnf1*, *sfa*, *pap*, *ial*, *lt*, *st*, *bfpA, eaeA, ipaH, iucC, fimA, stx1*, *stx2* and *ehly*) and were, therefore, considered *fimA*-negative, or the strains were *fimA*-positive, while still testing negative for all other virulence determinants. To assess presence of other genes of the *fimA* cluster, a *fimH* determinant, encoding adhesin mediating attachment of type 1 fimbriae, was tested on all *fimA*-positive strains. Except of 10 isolates, *fimA*-positive isolates were also positive for *fimH* determinants (98.3 % showed both determinants for type 1 fimbriae). Within groups of strains with different origins (Table 1), a relatively small proportion (≈17 %) of *E. coli* strains had no detected virulence determinants. Therefore, all strains were analyzed without regard for their origin.

**Table 1:** Origin of *E. coli* strains and proportion of *fimA*-negative or *fimA*-positive strains

Origin of *E. coli* strains	*fimA*-negative *E. coli* strains (*n* = 96)	*fimA*-positive *E. coli* strains (*n* = 483)	Total
University hospitals Brno	63 (15.8 %)	336 (84.2 %)	399 (100 %)
University teaching hospital Hradec Králové	9 (20.5 %)	35 (79.5 %)	44 (100 %)
Strains isolated from pigs	18 (18.0 %)	82 (82.0 %)	100 (100 %)
Strains isolated from non-human primates	6 (16.7 %)	30 (83.3 %)	36 (100 %)
Total	96 (16.7 %)	483 (83.4 %)	579 (100 %)

### Biochemical analysis

*E. coli* isolates (*n* = 579) were positive for the following biochemical reactions: TRE (99.1 %), MAN (99.0 %), SOR (97.8 %), ONP (97.6 %) IND (97.4 %), LYS (93.6 %), SUC (51.8 %), ORN (50.4 %), ESL (14.2 %), ADO (8.8 %), H_2_S (3.6 %), CEL (2.2 %), SCI (2.1 %), MAL (1.0 %), URE (0.7 %), INO (0.7 %) and PHE (0.2 %). The utilization of substrate showed variable results in ORN and SUC reactions between *fimA*-positive and *fimA*-negative *E. coli* strains. Biochemical analysis showed that the production of ornithine decarboxylase was significantly higher among *fimA*-positive *E. coli* strains (*n* = 254; 52.6 %) compared to *fimA*-negative strains (*n* = 38; 39.6 %, *p* = 0.03). In addition, production of succinate dehydrogenase was significantly higher among *fimA*-positive *E. coli* strains (*n* = 269; 55.7 %) compared to *fimA*-negative *E. coli* strains (*n* = 31; 32.3 %, *p* < 0.01).

### Detection of phylogenetic groups in *E. coli* strains

Phylogenetic analysis of 579 *E. coli* strains revealed that *fimA*-negative *E. coli* strains contained a significantly higher prevalence of phylogenetic group A (66.7 %) compared to *fimA*-positive *E. coli* strains (39.1 %; *p* < 0.01). Phylogroups B1 and B2 were found less frequently among *fimA*-negative *E. coli* strains (*p* = 0.01 and *p* = 0.04, respectively), while the prevalence of phylogroup D was similar in both groups of *E. coli* strains (Table 2). Frequency of bacteriocin production in *E. coli* strains belonging to phylogenetic groups A, B1, B2 and D, respectively, was not significantly different between *fimA*-positive and *fimA*-negative *E. coli* strains (Additional file 1: Table S1).

**Table 2:** Prevalence of phylogroups and bacteriocin determinants among *fimA*-negative and *fimA*-positive *E. coli* strains

Prevalence of phylogroups and bacteriocin determinants	*fimA*-negative *E. coli* strains (*n* = 96)	*fimA*-positive *E. coli* strains (*n* = 483)	*p*-value
Phylogroup A	64 (66.7 %)	189 (39.1 %)	*p* < 0.01
Phylogroup B1	8 (8.3 %)	92 (19.0 %)	*p* = 0.01
Phylogroup B2	9 (9.4 %)	89 (18.4 %)	*p* = 0.04
Phylogroup D	15 (15.6 %)	113 (23.4 %)	-
Bacteriocinogeny	21 (21.9 %)	172 (35.6 %)	*p* < 0.01
Microcin determinants	9 (9.4 %)	108 (22.4 %)	*p* < 0.01
Colicin determinants	25 (26.0 %)	187 (38.7 %)	*p* = 0.02
Colicin E1 determinants	1 (1.0 %)	39 (8.1 %)	*p* < 0.01

### Detection of bacteriocin-encoding determinants

Genetic determinants encoding 30 bacteriocin types including 23 colicins (A, B, D, E1, E2-9, Ia, Ib, Js, K, L, M, N, S4, U, Y and 5/10) and 7 microcins (H47, M, B17, C7, J25, L and V) were tested in all 579 *E. coli* strains used in this study. Strains with identified bacteriocin genes were more frequently found among *fimA*-positive *E. coli* strains (35.6 %) compared to *fimA*-negative *E. coli* strains (21.9 %, *p* < 0.01). Altogether, 3 microcin types and 8 colicin types were identified among *fimA*-negative *E. coli* strains while all 7 tested microcin types and 14 of the colicin types were found among *fimA*-positive *E. coli* strains (Tables 2 and 3). All of the identified bacteriocin determinants found among *fimA*-negative *E. coli* strains were also found among the *fimA*-positive *E. coli* strains.

**Table 3:** Distribution of bacteriocin encoding genes among *fimA*-negative and *fimA*-positive *E. coli* strains

Bacteriocin types	*fimA*-negative *E. coli* (%) (*n* = 96)	*fimA*-positive *E. coli* (%) (*n* = 483)
Colicin A	-	-
Colicin B	2 (2.1)	13 (2.7)
Colicin D	-	-
Colicin E1	1 (1.0)	39 (8.1)
Colicin E2	-	1 (0.2)
Colicin E3	-	-
Colicin E4	-	-
Colicin E5	-	-
Colicin E6	-	-
Colicin E7	2 (2.1)	2 (0.4)
Colicin E8	-	1 (0.2)
Colicin E9	-	-
Colicin Ia	10 (10.4)	56 (11.6)
Colicin Ib	2 (2.1)	32 (6.6)
Colicin K	1 (1.0)	3 (0.6)
Colicin L	-	-
Colicin M	6 (6.3)	30 (6.2)
Colicin N	-	2 (0.4)
Colicin S4	-	2 (0.4)
Colicin U	-	-
Colicin Y	-	2 (0.4)
Colicin 5/10	-	1 (0.2)
Colicin Js	1 (1.0)	3 (0.6)
Microcin B17	-	11 (2.3)
Microcin C7	-	2 (0.4)
Microcin H47	5 (5.2)	42 (8.7)
Microcin J25	-	1 (0.2)
Microcin L	-	1 (0.2)
Microcin M	1 (1.0)	33 (6.8)
Microcin V	3 (3.1)	18 (3.7)

A higher number of microcin determinants was found among *fimA*-positive *E. coli* strains (22.4 %) compared to *fimA*-negative *E. coli* strains (9.4 %; *p* < 0.01). Similarly, a higher number of detected colicin determinants was also found among *fimA*-positive *E. coli* strains (38.7 %) compared to *fimA*-negative *E. coli* strains (26.0 %; *p* = 0.02). In addition, an increased number of strains encoding colicin E1 was found among *fimA*-positive *E. coli* strains (8.1 %) compared to *fimA*-negative *E. coli* strains (1.0 %; *p* < 0.01).

### *In silico* analysis of *E. coli* genomes

A set of 1951 publicly available *E. coli* genomes including 121 completed genomes in the NCBI database was analyzed for the presence of virulence determinants tested in our study. Out of 1951 genomes, 490 genomes (25 %) met the criteria of our study (i.e. absence of tested virulence determinants or the sole presence of *fimA* determinant). In this set, 286 (58 %) genomes contained type 1 fimbriae as the only detected virulence determinant (*fimA*-positive *E. coli* strains) and 204 (42 %) genomes did not contain any of the 18 tested virulence factors (*fimA*-negative *E. coli* strains). No significant difference was found in the prevalence of bacteriocin determinants in the group of *fimA*-positive *E. coli* strains (15.4 %) compared to *fimA*-negative *E. coli* strains (14.7 %).

In addition, the subset of 121 complete genomes was analyzed. Out of them, 64 genomes were suitable for our study (i.e. contained no tested virulence determinants or contained only *fimA* determinant). While 50 (78 %) genomes belonged to *fimA-*positive group, 14 (22 %) genomes were *fimA*-negative. Bacteriocin genes were detected in only three *fimA*-positive genomes.

## Discussion

The *fimA*-negative *E. coli*, as well as fimbriae type I-possessing *E. coli* strains, used in this study were found to have similar frequencies in both humans and animals. A relatively small proportion (≈17 %) of *E. coli* strains had no detected virulence factors. These results are in accordance with other published data where 83 – 100 % of *fimA*-positive *E. coli* strains were found [7, 23]. Detection of both *fimA* and *fimH* determinants in majority of strains suggests that the complete *fimA* cluster is present in most of the tested strains.

*In silico* analysis of 121 complete genomes identified *fimA* determinant in 78 % of *E. coli* complete genomes. This finding is in accordance to previous studies [7, 23] and also with the experimental results of this work, where *fimA* virulence determinant was identified in more than 80 % of isolates. On the other hand, the *fimA* determinant was identified only in 25 % of 1830 draft genomes suggesting that the *fimA* determinant likely remained unsequenced in a number of draft genomes. Similar underrepresentation was found also for bacteriocin determinants (24 % and 38 % of all complete and draft genomes contained bacteriocin determinants, respectively; data not shown). This is in contrast to experimentally determined prevalence of bacteriocinogeny among human *E. coli* isolates where over 50 % of *E. coli* strains produced bacteriocins [17, 24]. These findings suggest that *in silico* analysis of draft genomes is of limited value in this and similar studies.

To assess clonal character of isolates, the obtained data from biochemical screening and analysis of phylogenetic groups and bacteriocin determinants were analyzed using Paup^*^ 4 (Phylogenetic Using Analysis Parsimony). Using this approach, 52 and 226 individual strain types (data not shown) were identified in the groups of 96 and 483 *fimA*-negative *E. coli* isolates and fimbriae type I-possessing *E. coli* isolates, respectively, indicating that *E. coli* isolates in this study were not predominantly of clonal character.

*E. coli* type 1 fimbriae mediate adhesion to a number of host cell types including epithelial, endothelial and lymphoid cells [10, 25, 26], where they recognize mannose-containing glycoproteins and activate epithelial cells via Toll-like receptor 4 [27]. Type 1 fimbriae are expressed by both uropathogenic and fecal *E. coli* strains. In murine models, type 1 fimbriae have been shown to be important in the persistence of *E. coli* urine infections and deletion of the *fim* gene cluster from the virulent *E. coli* strain O1:K1:H7 has been shown to decrease the virulence of this strain in the urinary tract infection model [22, 28]. However, several other studies have demonstrated that the presence of the *fim* gene cluster was not correlated with uropathogenicity in humans [21, 29–33]. Additionally, *E. coli* strain A0 34/86 (O83:K24:H31), which has been approved as live oral vaccine preparation for infants in the Czech and Slovak Republic, was shown to possess type 1 fimbriae [34, 35]. Another widely used probiotic strain, *E. coli* Nissle 1917, is known to possess type 1 fimbriae as well as other adhesins (e.g. F1C fimbriae) [36, 37]. These examples demonstrate that type 1 fimbriae are primarily important for attachment to eukaryotic cells and could be, in certain strains, important also with regard to virulence.

While phylogroup A (and also B1) consists mostly of commensal strains, phylogroup B2 (and also D) consists mainly of extraintestinal pathogenic *E. coli* strains [4–6, 38]. The majority of tested strains in this study (61 %) belonged to A and B1 phylogenetic groups, which was the direct result of sampling *E. coli* strains without a specific set of virulence factors or *E. coli* strains harboring *fimA* determinant as the only detected virulence gene. These findings are in agreement with the observation that non-pathogenic *E. coli* strains are usually in phylogroup A and B1 [1, 39]. Interestingly, our set of strains also contained strains from group B2 (17 %). Moreover, no virulence factors were detected in of the 9 strains from this phylogroup, which indicates that the relationship between *E. coli* phylogroup and the presence of virulence factors is not exclusive.

Since prevalence of phylogroups among *fimA*-negative strains differed from *fimA*-positive *E. coli* strains, it is possible that the observed differences in the prevalence of bacteriocin determinants between both groups of *E. coli* strains were in fact due to differences in the *E. coli* phylogroups. However, there is no clear association between bacteriocinogeny and *E. coli* phylogroups. Gordon and O'Brien (2006) detected 4 phylogenetic groups and 19 bacteriocin types in a set of 266 fecal *E. coli* strains and did not find significant differences in the frequency of bacteriocinogeny in different *E. coli* phylogroups [40]. In our previous study, we have found that prevalence of colicinogenic strains was higher in phylogroups A and D compared to phylogroups B2 [17]. In contrast, the study of Budič et al. (2011) revealed increased bacteriocinogeny in the phylogroup B2 among 105 uropathogenic strains [18]. In this study, no differences in the prevalence of bacteriocinogeny were found among *fimA*-negative *E. coli*, while an increased bacteriocinogeny was found in the phylogenetic group B2 compared to phylogenetic group A in the set of *fimA-*positive *E. coli* strains. Although more frequent phylogenetic group B2 could be the reason of increased prevalence of bacteriocinogeny among *fimA*-positive *E. coli* strains, increased prevalence of bacteriocin genes were found in all tested *E. coli* phylogroups (statistically not significant; Additional file 1: Table S1), suggesting the association between bacteriocinogeny and the *fimA* gene cluster.

In humans, two types of commensal *E. coli* strains (resident and transient) are known to exist. They differ in their ability to persist in the human intestine. While resident strains are present in the intestines of an individual for months at a time, transient strains only persist for days to weeks [41–43]. In addition, it has been shown that the ability of *E. coli* strains to persist in the human intestines is associated with several virulence factors, especially various fimbriae [44, 45]. Since the *E. coli* strains in phylogroup B2 are typical for resident flora [39, 46, 47] and *E. coli* of phylogroup A is typical for transient strains [39] the *fimA*-negative *E. coli* isolates in this study appear to be more frequently transient strains.

The *fimA*-positive *E. coli* strains were more often positive for activity of ornithine decarboxylase compared to *fimA*-negative *E. coli.* Activity of ornithine decarboxylase, which results in production of polyamines (e.g. putrescine), helps to cope with stress conditions, such as oxidative radicals [48] and low pH [49]. In addition, polyamines play an important role in biofilm formation [50]. There is a relationship between cellular adherence and biofilm formation in certain strains of *E. coli* [51].

This study has shown that fecal *fimA*-positive *E. coli* strains produced bacteriocins more often compared to similar, but *fimA*-negative, strains. Bacteriocin synthesis appears to be important in microbial communities because of its potential invasive and defensive roles [52]. Moreover, antimicrobial effect of individual bacteriocin types showed differences with respect to their activity on *E. coli* strains [18]. In previous studies, the occurrence of several bacteriocin genes was found to be associated with several genes encoding virulence factors [16–20] and the results of this study extends the original findings. Bacteriocin types and their sequences have been shown to be host population-specific [53], indicating that bacteriocin-encoding determinants mainly spread among and within hosts. Since virulence genes likely evolved and are being maintained to improve inter-host persistence of commensal bacteria [54, 55], bacteriocin synthesis may further promote stable colonization of the gut. Similar findings were published by Gillor et al. 2009 [56], in which they reported that bacteriocinogeny plays a significant role in the colonization of *E. coli* in the intestinal tract. As with type 1 fimbriae, which were shown to increase virulence in the urinary tract infection model [26, 27], synthesis of colicin E1 was found to be associated with uropathogenic strains [17].

## Conclusions

In summary, *fimA*-positive *E. coli* strains of human and animal origin were found most often to be in phylogroup B2; additionally, *fimA*-positive *E. coli* strains tested positive for ornithine decarboxylase, succinate dehydrogenase and bacteriocin synthesis more frequently than *fimA*-negative *E. coli* strains. All these findings are consistent with increased adherence to intestinal epithelium, increased bacterial virulence, and increased ability to survive in the intestine.

## Methods

### Bacterial strains

The origins of *E. coli* strains used in this study are shown in Table 1. *E. coli* strains were collected between 2007 and 2012 from intestinal microflora of patients at two University Hospitals in Brno (*n* = 399) and one University Teaching Hospital in Hradec Králové (*n* = 44), Czech Republic. Strains were collected from feces of patients without bacterial gut infection. The patients were admitted for a number of concerns including infectious and parasitic diseases (*n* = 165); neoplasms (*n* = 60); blood diseases (*n* = 2); endocrine, nutritional and metabolic diseases (*n* = 42); mental and behavioral disorders (*n* = 4); diseases of the nervous system (*n* = 5); diseases of the circulatory system (*n* = 7); diseases of the respiratory system (*n* = 4); diseases of the digestive system (*n* = 73); diseases of the skin and subcutaneous tissue (*n* = 5); diseases of the musculoskeletal system and connective tissue (*n* = 3); diseases of the genitourinary system (*n* = 6); symptoms, signs and abnormal clinical and laboratory findings, not elsewhere classified (*n* = 30); injury, poisoning and certain other consequences of external causes (*n* = 5); and factors influencing health status and contact with health services (*n* = 32). An International Statistical Classification of Diseases and Related Health Problems, 10th Revision (ICD-10)-2015-WHO Version for 2015, was used for the classification of diseases. In addition, fecal *E. coli* strains of animal origin were isolated from pigs (*n* = 100) and non-human primates (NHP) (*n* = 36). The animal isolates were included into the study because of their availability and because there was no statistically significant difference in the ratio of *fimA*-negative to *fimA*-positive strains between human and animal *E. coli* strains. *E. coli* strains from pigs were isolated during 2010–2012 in Hradec Králové [57, 58]. *E. coli* strains isolated from NHP feces were collected in 2012 from 7 zoological gardens in the Czech and Slovak Republic (Zoological Garden Hodonín (48°51′52.06″N, 17°6′24.52″E), Zoological Garden Jihlava (49°23'51.834"N, 15°35'57.872"E), Zoological Garden Košice (48°47'00.8"N, 21°12'13.6"E), Zoological Garden Liberec (50°46′34.038″N, 15°4′32.655″E), Zoological and Botanical Garden Plzeň (49°45'27.85"N, 13°21'35.90″E), The Prague Zoological Garden (50°7'0.099"N, 14°24'39.676"E) and Zoological Garden Zlín - Lešná (49°16'20.048"N, 17°42'54.118"E)). From each patient or animal, a single *E. coli* strain was isolated using selective diagnostic ENDO agar. Metabolic profiles of isolates were obtained during determination of *E. coli* among isolates using commercial screening kit EnteroTest 16 (test for the presence of several metabolic reactions (H_2_S, LYS, IND, ORN, URE, PHE, ESL, SCI, MAL, INO, ADO, CEL, SUC, SOR, TRE and MAN) (Lachema, Brno, CZ) and ONP test for detection of β-galactosidase (Lachema, Brno, CZ). The obtained metabolic profiles were compared with the database (TNW ProAuto 7 software) for classification of isolates.

All human data used in the study were anonymized and the study was approved by the Joint Ethical Committee (Charles University in Praha, Faculty of Medicine at Hradec Králové & University Teaching Hospital Hradec Králové) and the ethics committee of the Faculty of Medicine, Masaryk University, Czech Republic. All clinical samples were collected after patients gave written informed consent for participation in the study and for their samples to be used for research. For children under the age of 18, consent was obtained from parents. The animal part of the study (i.e. *E. coli* strains isolated from pigs) was approved by the Institutional Review Board of the Animal Care Committee of the Institute of Experimental Biopharmaceutics, Academy of Sciences of the Czech Republic, Record Number 1492006. NHP fecal samples were collected after presentation of a preliminary research plan that specified the agreement between particular ZOO zoologists or veterinarians. We obtained all the required permits needed to collect the samples, which were collected during routine cages cleaning, without direct contact or interaction with animals.

### Detection of virulence determinants

*E. coli* strains were tested for the presence of 18 virulence determinants (*α*-*hly*, *afaI*, *aer*, *cnf1*, *sfa*, *pap*, pCVD432, *ial*, *lt*, *st*, *bfpA*, *eaeA*, *ipaH*, *iucC*, *fimA*, *stx1*, *stx2* and *ehly*). Primer pair sequences and PCR product lengths are shown in Additional file 2: Table S2; the PCR protocols were previously described [59–67]. Positive controls for detection of virulence genes were taken from the laboratory stock and comprised following strains: *E. coli* B2917 (*pCVD432*), *E. coli* B3428 (*α-hly*), *E. coli* B3406 (*afaI*), *E. coli* B3427 (*aer*), *E. coli* B3410 (*cnf1*), *E. coli* B3418 (*sfa*), *E. coli* B3406 (*pap*), *E. coli* B3430 (*ial*), *E. coli* B2541 (*st*), *E. coli* B2802 (*lt*), *E. coli* B1804 (*bfpA*), *E. coli* B2905 (*eaeA*), *E. coli* B2987 (*ipaH*), *E. coli* B3411 (*iucC*), *E. coli* B3404 (*aer*), *E. coli* B3423 (*fimA*) and *E. coli* B2871 (*ehly*). To assess presence of other genes of the *fimA* cluster, a *fimH* determinant, encoding adhesin mediating attachment of type 1 fimbriae, was tested on a set of *fimA*-positive strains. *E. coli* B3423 strain was used as a positive control for *fimH* gene. *E. coli* strains with none of the 18 tested virulence determinants were used as control strains (i.e. *fimA*-negative *E. coli* strains), while the experimental strains consisted of *E. coli* strains encoding only fimbriae type I (i.e. *fimA*-positive *E. coli* strains).

### Detection of bacteriocinogeny and bacteriocin determinants

*E. coli* strains were cultivated (37 °C for 48 h) in parallel on (i) TY agar and (ii) nutrient broth agar plates. The TY agar consisted of yeast extract (Hi-Media, Mumbai, India) 5 gl^−1^, tryptone (Hi-Media) 8 gl^−1^, sodium chloride 5 gl^−1^, and a 1.5 % (w/v) of agar (Hi-Media). Nutrient broth agar contained a Nutrient Agar (HiMedia) 28 gl^−1^. The bacteria were then killed using chloroform vapors and each plate was then overlaid with a thin layer of soft TY agar (0.7 %; w/v) containing 10^7^ cells ml^−1^ of an indicator strain. The plates were then incubated at 37 °C overnight and bacteriocin producers were identified [17, 20]. Indicator strains *E. coli* K12-Row, C6 (ϕ), B1, P400, and *Shigella sonnei* 17 and *E. coli* S40 were used to detect bacteriocin production [17, 20]. The set of these strains is capable to detect all known colicin types and most of the microcin types.

Altogether, 30 bacteriocin types were detected among tested strains (23 colicin and 7 microcin genes) using methods previously described [17, 20, 40]. Isolated DNA (using DNAzol reagent, Invitrogen, Carlsbad, CA, according to the manufacturer's protocol) was diluted 100-fold in sterile distilled water. Alternatively, one bacterial colony of each *E. coli* strain was resuspended in 100 μl of sterile distilled water and 1 μl of this suspension was added to the PCR mix. A list of primers is shown in Additional file 2: Table S2. Cycling conditions were 94 °C (2 min); 94 °C (30 s), 60 °C (30 s), 72 °C (1 min), 30 cycles; and 72 °C (7 min). For colony PCR, the initial step was set for 5 min. For identification of bacteriocin determinants among tested strains, known bacteriocin producers were used as positive controls: *E. coli* BZB2101pColA - CA31, BZB2102 pColB - K260, BZB2103 pColD - CA23, BZB2107 pColE4 - CT9, BZB2108 pColE5 - 099, BZB2150 pColE6 - CT14, BZB2120 pColE7 - K317, BZB2279 pColIa - CA53, BZB2202 ColIb - P9, BZB2116 pColK - K235, PAP1 pColM - BZBNC22, BZB2123 pColN - 284 (original source: A. P. Pugsley), *E. coli* 189BM pColE2 - P9 (B. A. D. Stocker), *E. coli* 385/80 pColE1, pColV (H. Lhotová), *E. coli* 185 M4 pColE3 - CA38 (P. Fredericq), *E. coli* W3110 pColE8, W3110 pColE9 (J. R. James), *E. coli* K-12 pColS4 (D. Šmajs), *S. boydii* M592 (serovar 8) pColU (V. Horák), *E. coli* K339 pColY (D. Friedman), *Shigella sonnei* (colicinotype 7) pColJs (J. Šmarda), *E. coli* pCol5 and *E. coli* pCol10 (H. Pilsl). As microcin control producers, the following bacterial strains were used: *E. coli* 449/82 pColX (microcin B17); *E. coli* 313/66 pColG (microcin H47); *E. coli* 363/79 pColV (microcin V, original source: H. Lhotová); *E. coli* TOP10F' pDS601 (microcin C7); *E. coli* D55/1 (microcin J25); *E. coli* B1239 (microcin L, D. Šmajs). *E. coli* B3423 strain was used as a positive control for *fimH* gene detection. Because of sensitivity of microcins H47 and M to chloroform vapours, all *E. coli* strains were tested by PCR method for the presence of mH47 and mM genes [36]. PCR products of related bacteriocin types (colicins E2-9, Ia-Ib, U-Y) were sequenced using dideoxy-terminator sequencing with amplification primers. Sequence analyses were carried out using Lasergene software (DNASTAR, Inc., Madison, WI).

### Phylogenetic analysis of *E. coli* strains

A previously described triplex PCR method [68] was used to assign *E. coli* strains to one of four main phylogenetic groups (A, B1, B2 and D).

### Statistical analyses

The statistical analyses of the prevalence bacteriocin and phylogroups used standard methods derived from the binomial distribution, including the two-tailed Fisher's exact test. *STATISTICA* software, version 8.0 (StatSoft, Tulsa, OK), was used for calculations.

### *In silico* analysis of *E. coli* genomes

In total, 121 complete and 1830 draft genome sequences of *E. coli* strains were downloaded as FASTA files from ftp://ftp.ncbi.nlm.nih.gov/genomes/Bacteria/ and ftp://ftp.ncbi.nlm.nih.gov/genomes/Bacteria_DRAFT/ NCBI public databases, respectively, using ncbi_ftp_download script (available at: https://github.com/aleimba/bac-genomics-scripts/). For determination of the presence/absence of virulence determinants and bacteriocin encoding genes in downloaded genome sequences, DNA comparison using Smith-Waterman algorithm [69], implemented in a cross-match software (unpublished) was used. Identity scores higher than 75 % were used.

## Availability of supporting data

The data set supporting the results of this article is included in the Additional file 1: Table S1. The data set of colicin gene sequences supporting the results of the article has been deposited in the GenBank/EMBL/DDBJ. Accession numbers for colicin sequences are shown in the Additional file 3: Table S3.

## Additional files

Additional file 1: Table S1. Complete data set presented in this article. (DOCX 19 kb) Additional file 2: Table S2. DNA primers used for PCR detection of colicin and microcin encoding genes and genes encoding virulence factors. (XLSX 167 kb) Additional file 3: Table S3. Colicin gene sequences deposited in the GenBank/EMBL/DDBJ. (XLSX 19 kb)

## Abbreviations

*E. coli*: *Escherichia coli*
TRE: Trehalose
MAN: Mannitol
SOR: Sorbitol
ONP: Beta-galatosidase
IND: Indole
LYS: Lysine
SUC: Sucrose
ORN: Ornithine
ESL: Esculin
ADO: Adonitol
H_2_S: Hydrogen sulphide
CEL: Cellobiose
SCI: Simmons citrate
MAL: MAL
URE: Urease
INO: Inositol
PHE: Phenylalanine

## References

[1] Tenaillon O, Skurnik D, Picard B, Denamur E (2010). The population genetics of commensal *Escherichia coli*. Nat Rev Microbiol.

[2] Bergthorsson U, Ochman H (1998). Distribution of chromosome length variation in natural isolates of *Escherichia coli*. Mol Biol Evol.

[3] Dobrindt U, Agerer F, Michaelis K, Janka A, Buchrieser C, Samuelson M, Svanborg C, Gottschalk G, Karch H, Hacker J (2003). Analysis of genome plasticity in pathogenic and commensal *Escherichia coli* isolates by use of DNA arrays. J Bacteriol.

[4] Boyd EF, Hartl DL (1998). Chromosomal regions specific to pathogenic isolates of *Escherichia coli* have a phylogenetically clustered distribution. J Bacteriol.

[5] Picard B, Garcia JS, Gouriou S, Duriez P, Brahimi N, Bingen E, Elion J, Denamur E (1999). The link between phylogeny and virulence in *Escherichia coli* extraintestinal infection. Infect Immun.

[6] Johnson JR, Stell AL (2000). Extended virulence genotypes of *Escherichia coli* strains from patients with urosepsis in relation to phylogeny and host compromise. J Infect Dis.

[7] Kaczmarek A, Budzynska A, Gospodarek E (2012). Prevalence of genes encoding virulence factors among *Escherichia coli* with K1 antigen and non-K1 *E. coli* strains. J Med Microbiol.

[8] Klemm P, Jørgensen BJ, van Die I, de Ree H, Bergmans H (1985). The *fim* genes responsible for synthesis of type 1 fimbriae in *Escherichia coli*, cloning and genetic organization. Mol Gen Genet.

[9] Jones CH, Pinkner JS, Roth R, Heuser J, Nicholes AV, Abraham SN, Hultgren SJ (1995). FimH adhesin of type 1 pili is assembled into a fibrillar tip structure in the *Enterobacteriaceae*. Proc Natl Acad Sci USA.

[10] Khan NA, Kim Y, Shin S, Kim KS (2007). FimH-mediated *Escherichia coli* K1 invasion of human brain microvascular endothelial cells. Cell Microbiol.

[11] Braun V, Pilsl H, Gross P (1994). Colicins: structures, modes of action, transfer through membranes, and evolution. Arch Microbiol.

[12] Šmarda J, Šmajs D (1998). Colicins--exocellular lethal proteins of *Escherichia coli*. Folia Microbiol (Praha).

[13] Cascales E, Buchanan SK, Duché D, Kleanthous C, Lloubès R, Postle K, Riley M, Slatin S, Cavard D (2007). Colicin biology. Microbiol Mol Biol Rev.

[14] Šmajs D, Weinstock GM (2001). Genetic organization of plasmid ColJs, encoding colicin Js activity, immunity, and release genes. J Bacteriol.

[15] Šmajs D, Weinstock GM (2001). The iron- and temperature-regulated *cjrBC* genes of *Shigella* and enteroinvasive *Escherichia coli* strains code for colicin Js uptake. J Bacteriol.

[16] Azpiroz MF, Poey ME, Laviña M (2009). Microcins and urovirulence in *Escherichia coli*. Microb Pathog.

[17] Šmajs D, Micenková L, Šmarda J, Vrba M, Ševčíková A, Vališová Z, Woznicová V (2010). Bacteriocin synthesis in uropathogenic and commensal *Escherichia coli*: colicin E1 is a potential virulence factor. BMC Microbiol.

[18] Budič M, Rijavec M, Petkovšek Z, Zgur-Bertok D (2011). *Escherichia coli* bacteriocins: antimicrobial efficacy and prevalence among isolates from patients with bacteraemia. PloS One.

[19] Petkovšek Z, Zgur-Bertok D, Starcic Erjavec M (2012). Colicin insensitivity correlates with a higher prevalence of extraintestinal virulence factors among *Escherichia coli* isolates from skin and soft-tissue infections. J Med Microbiol.

[20] Micenková L, Štaudová B, Bosák J, Mikalová L, Littnerová S, Vrba M, Ševčíková A, Woznicová V, Šmajs D (2014). Bacteriocin-encoding genes and ExPEC virulence determinants are associated in human fecal *Escherichia coli* strains. BMC Microbiol.

[21] Johnson JR (1991). Virulence factors in *Escherichia coli* urinary tract infection. Clin Microbiol Rev.

[22] Connell I, Agace W, Klemm P, Schembri M, Mărild S, Svanborg C (1996). Type 1 fimbrial expression enhances *Escherichia coli* virulence for the urinary tract. Proc Natl Acad Sci USA.

[23] Gonçalves A, Igrejas G, Radhouani H, Santos T, Monteiro R, Pacheco R, Alcaide E, Zorrilla I, Serra R, Torres C, Poeta P (2013). Detection of antibiotic resistant enterococci and *Escherichia coli* in free range Iberian Lynx (*Lynx pardinus*). Sci Total Environ.

[24] Šmarda J, Obdrzálek V (2001). Incidence of colicinogenic strains among human *Escherichia coli*. J Basic Microbiol.

[25] Abraham S, Shin J, Malaviya R (2001). Type 1 fimbriated *Escherichia coli*-mast cell interactions in cystitis. J Infect Dis.

[26] Ponniah S, Abraham SN, Dockter ME, Wall CD, Endres RO (1989). Mitogenic stimulation of human B lymphocytes by the mannose-specific adhesin on *Escherichia coli* type 1 fimbriae. J Immunol.

[27] Hedlund M, Frendéus B, Wachtler C, Hang L, Fischer H, Svanborg C (2001). Type 1 fimbriae deliver an LPS- and TLR4-dependent activation signal to CD14-negative cells. Mol Microbiol.

[28] Mulvey MA, Schilling JD, Hultgren SJ (2001). Establishment of a persistent *Escherichia coli* reservoir during the acute phase of a bladder infection. Infect Immun.

[29] Hagberg L, Jodal U, Korhonen TK, Lidin-Janson G, Lindberg U, Svanborg Edén C (1981). Adhesion, hemagglutination, and virulence of *Escherichia coli* causing urinary tract infections. Infect Immun.

[30] Leffler H, Svanborg-Edén C (1981). Glycolipid receptors for uropathogenic *Escherichia coli* on human erythrocytes and uroepithelial cells. Infect Immun.

[31] Edén CS, Freter R, Hagberg L, Hull R, Hull S, Leffler H, Schoolnik G (1982). Inhibition of experimental ascending urinary tract infection by an epithelial cell-surface receptor analogue. Nature.

[32] Väisänen-Rhen V, Elo J, Väisänen E, Siitonen A, Orskov I, Orskov F, Svenson SB, Mäkelä PH, Korhonen TK (1984). P-fimbriated clones among uropathogenic *Escherichia coli* strains. Infect Immun.

[33] Bergsten G, Wullt B, Svanborg C (2005). *Escherichia coli*, fimbriae, bacterial persistence and host response induction in the human urinary tract. Int J Med Microbiol.

[34] Lodinová R, Jouja V, Vinsová N, Vocel J, Melková J (1980). New attempts and possibilities in prevention and treatment of intestinal coli-infections in infants. Czech Med.

[35] Lodinová-Zádníková R, Tlaskalová H, Korych B, Bartáková Z (1995). The antibody response in infants after oral administration of inactivated and living *E. coli* vaccines and their protective effect against nosocomial infections. Adv Exp Med Biol.

[36] Patzer SI, Baquero MR, Bravo D, Moreno F, Hantke K (2003). The colicin G, H and X determinants encode microcins M and H47, which might utilize the catecholate siderophore receptors FepA, Cir, Fiu and IroN. Microbiol Read Engl.

[37] Grozdanov L, Raasch C, Schulze J, Sonnenborn U, Gottschalk G, Hacker J, Dobrindt U (2004). Analysis of the genome structure of the nonpathogenic probiotic *Escherichia coli* strain Nissle 1917. J Bacteriol.

[38] Bingen E, Picard B, Brahimi N, Mathy S, Desjardins P, Elion J, Denamur E (1998). Phylogenetic analysis of *Escherichia coli* strains causing neonatal meningitis suggests horizontal gene transfer from a predominant pool of highly virulent B2 group strains. J Infect Dis.

[39] Nowrouzian FL, Wold AE, Adlerberth I (2005). *Escherichia coli* strains belonging to phylogenetic group B2 have superior capacity to persist in the intestinal microflora of infants. J Infect Dis.

[40] Gordon DM, O'Brien CL (2006). Bacteriocin diversity and the frequency of multiple bacteriocin production in *Escherichia coli*. Microbiology.

[41] Sears HJ, Brownlee I, Uchiyama JK (1950). Persistence of individual strains of *Escherichia coli* in the intestinal tract of man. J Bacteriol.

[42] Sears HJ, Brownlee I (1952). Further observations on the persistence of individual strains of *Escherichia coli* in the intestinal tract of man. J Bacteriol.

[43] Sears HJ, Janes H, Saloum R, Brownlee I, Lamoreaux LF (1956). Persistence of individual strains of *Escherichia coli* in man and dog under varying conditions. J Bacteriol.

[44] Adlerberth I, Svanborg C, Carlsson B, Mellander L, Hanson LA, Jalil F, Khalil K, Wold AE (1998). P fimbriae and other adhesins enhance intestinal persistence of *Escherichia coli* in early infancy. Epidemiol Infect.

[45] Nowrouzian F, Adlerberth I, Wold AE (2001). P fimbriae, capsule and aerobactin characterize colonic resident *Escherichia coli*. Epidemiol Infect.

[46] Zhang L, Foxman B, Marrs C (2002). Both urinary and rectal *Escherichia coli* isolates are dominated by strains of phylogenetic group B2. J Clin Microbiol.

[47] Gordon DM, Stern SE, Collignon PJ (2005). Influence of the age and sex of human hosts on the distribution of *Escherichia coli* ECOR groups and virulence traits. Microbiol Read Engl.

[48] Tkachenko AG, Akhova AV, Shumkov MS, Nesterova LY (2012). Polyamines reduce oxidative stress in *Escherichia coli* cells exposed to bactericidal antibiotics. Res Microbiol.

[49] Viala JPM, Méresse S, Pocachard B, Guilhon AA, Aussel L, Barras F (2011). Sensing and adaptation to low pH mediated by inducible amino acid decarboxylases in *Salmonella*. PloS One.

[50] Wortham BW, Oliveira MA, Fetherston JD, Perry RD (2010). Polyamines are required for the expression of key Hms proteins important for *Yersinia pestis* biofilm formation. Environ Microbiol.

[51] Puttamreddy S, Minion FC (2011). Linkage between cellular adherence and biofilm formation in *Escherichia coli* O157:H7 EDL933. FEMS Microbiol Lett.

[52] Riley MA, Wertz JE (2002). Bacteriocin diversity: ecological and evolutionary perspectives. Biochimie.

[53] Šmajs D, Čejková D, Micenková L, Lima-Bittencourt CI, Chartone-Souza E, Šmarda J, Nascimento AM (2012). Human *Escherichia coli* strains of different geographical and time source: bacteriocin types and their gene sequences are population-specific. Environ Microbiol Rep.

[54] Levin BR (1996). The evolution and maintenance of virulence in microparasites. Emerg Infect Dis.

[55] Le Gall T, Clermont O, Gouriou S, Picard B, Nassif X, Denamur E, Tenaillon O (2007). Extraintestinal virulence is a coincidental by-product of commensalism in B2 phylogenetic group *Escherichia coli* strains. Mol Biol Evol.

[56] Gillor O, Giladi I, Riley MA (2009). Persistence of colicinogenic *Escherichia coli* in the mouse gastrointestinal tract. BMC Microbiol.

[57] Bureš J, Šmajs D, Květina J, Förstl M, Šmarda J, Kohoutová D, Kuneš M, Tacheci I, Rejchrt S, Lesná J, Vorisek V, Kopáčová M (2011). Bacteriocinogeny in experimental pigs treated with indomethacin and *Escherichia coli* Nissle. World J Gastroenterol WJG.

[58] Šmajs D, Bureš J, Šmarda J, Chaloupková E, Květina J, Förstl M, Kohoutová D, Kuneš M, Rejchrt S, Lesná J, Kopáčová M (2012). Experimental administration of the probiotic Escherichia coli strain Nissle 1917 results in decreased diversity of *E. coli* strains in pigs. Curr Microbiol.

[59] Yamamoto S, Terai A, Yuri K, Kurazono H, Takeda Y, Yoshida O (1995). Detection of urovirulence factors in *Escherichia coli* by multiplex polymerase chain reaction. FEMS Immunol Med Microbiol.

[60] Schmidt H, Knop C, Franke S, Aleksic S, Heesemann J, Karch H (1995). Development of PCR for screening of enteroaggregative *Escherichia coli*. J Clin Microbiol.

[61] López-Saucedo C, Cerna JF, Villegas-Sepulveda N, Thompson R, Velazquez FR, Torres J, Tarr PI, Estrada-García T (2003). Single multiplex polymerase chain reaction to detect diverse loci associated with diarrheagenic *Escherichia coli*. Emerg Infect Dis.

[62] Kuhnert P, Hacker J, Mühldorfer I, Burnens AP, Nicolet J, Fey J (1997). Detection system for *Escherichia coli*-specific virulence genes: absence of virulence determinants in B and C strains. Appl Environ Microbiol.

[63] Martínez JL, Herrero M, de Lorenzo V (1994). The organization of intercistronic regions of the aerobactin operon of pColV-K30 may account for the differential expression of the *iucABCD iutA* genes. J Mol Biol.

[64] Paton AW, Paton JC (1998). Detection and characterization of Shiga toxigenic *Escherichia coli* by using multiplex PCR assays for *stx*_1_, *stx*_2_, *eaeA*, enterohemorrhagic *E. coli hlyA*, *rfb*_O111_, and *rfb*_O157_. J Clin Microbiol.

[65] Asadi KM, Oloomi M, Habibi M, Bouzari S (2012). Cloning of fimH and fliC and expression of the fusion protein FimH/FliC from Uropathogenic *Escherichia coli* (UPEC) isolated in Iran. Iran J Microbiol.

[66] Paciorek J (2002). Virulence properties of *Escherichia coli* faecal strains isolated in Poland from healthy children and strains belonging to serogroups O18, O26, O44, O86, O126 and O127 isolated from children with diarrhoea. J Med Microbiol.

[67] Bírošová E, Siegfried L, Kmetová M, Makara A, Ostró A, Gresová A, Urdzík P, Liptáková A, Molokácová M, Bártl R, Valanský L (2004). Detection of virulence factors in alpha-haemolytic *Escherichia coli* strains isolated from various clinical materials. Clin Microbiol Infect Off Publ Eur Soc Clin Microbiol Infect Dis.

[68] Clermont O, Bonacorsi S, Bingen E (2000). Rapid and simple determination of the *Escherichia coli* phylogenetic group. Appl Environ Microbiol.

[69] Smith TF, Waterman MS (1981). Identification of Common Molecular Subsequences. J Mol Biol.

