# Supplementary material for: Determinants encoding fimbriae type 1 in fecal Escherichia coli are associated with increased frequency of bacteriocinogeny
Source: BMC Microbiol. 2015 Oct 6;15:201. doi: 10.1186/s12866-015-0530-5 (PMC4594643; doi:10.1186/s12866-015-0530-5)
Supplement: Additional file 1: Table S1. — Complete data set presented in this article. (DOCX 19 kb) [file 12866_2015_530_MOESM1_ESM.docx]

**Table S1. DNA Primers used for PCR detection of colicin and microcin encoding genes and genes encoding virulence factors**

| **Bacteriocin types and virulence factors** | **Primer name** | **5´-sequence-3´** | **Length** | **Reference of primers** |
| --- | --- | --- | --- | --- |
|  |  |  | **of PCR product (nt)** |  |
| A | ColA-F | cgtggggaaaagtcatcatc |  |  |
|  | ColA-R | gctttgctctttcctgatgc | 475 | [17] |
| B | colicinB-F | aagaaaatgacgagaagacg |  |  |
|  | colicinB-R | gaaagaccaaaggctataagg | 492 | [34] |
| D | ColD-F | ctggactgctgctggtgata |  |  |
|  | ColD-R | gaaggtgcgcctactactgc | 420 | [17] |
| E1 | colicinE1-F | tgtggcatcgggcgagaata |  |  |
|  | colicinE1-R | ctgcttcctgaaaagcctttt | 649 | [34] |
| E1* | cea2F | ggtggaactggaggtagcaa |  |  |
|  | cea2R | acgtcgttgttgttctgcttcct | 357 | [20] |
| E2 | ColE2-F | tgatgctgctgcaaaagag |  |  |
|  | ColE2-R | ttcaaagcgttccctaccac | 409 | [17] |
| E3 | ColE3-F | taagcaggctgcatttgatg |  |  |
|  | ColE3-R | tcggatctggacctttcaac | 413 | [17] |
| E4 | ColE4-F | gaaggctgcatttgatgct |  |  |
|  | ColE4-R | cggatccggacctttaattt | 409 | [17] |
| E5 | ColE3-F | taagcaggctgcatttgatg |  |  |
|  | ColE5-R | ttgaattctcgaatcgtcca | 430 | [17] |
| E6 | ColE6-F | accgaacgtccaggtgtt |  |  |
|  | ColE6-R | tttagcctgtcgctcctgat | 399 | [17] |
| E7 | ColE7-F | gcattctgccatctgaaat |  |  |
|  | ColE7-R | cttctgcccactttctttcg | 431 | [17] |
| E8 | ColE3-F | taagcaggctgcatttgatg |  |  |
|  | ColE8-R | gactgattggcttgtcgtga | 449 | [17] |
| E9 | ColE3-F | taagcaggctgcatttgatg |  |  |
|  | ColE9-R | gacttttctccctccgacct | 418 | [17] |
| Ia | ColIa-F | gcatgcaaatgacgctctta |  |  |
|  | ColIa-R | gaggacgccagttctctgtc | 473 | [17] |
| Ib | ColIb-F | aacgagtgggtcgatgattc |  |  |
|  | ColIb-R | ccttttctgcgctcgtattc | 464 | [17] |
| Js | ColJs-F | tcaaaatgtttgggctcctc |  |  |
|  | ColJs-R | taatctgccctgtcccactg | 254 | [17] |
| K | ColK-F | cagaggtcgctgaacatgaa |  |  |
|  | ColK-R | tccgctaaatcctgagcaat | 469 | [17] |
| L | Col28b(L)-F | tgcatattgaaagcgtcagc |  |  |
|  | Col28b(L)-R | caggttatcccctctcacca | 449 | [20] |
| M | ColM-F | gcttaccacttcgcaaaacc |  |  |
|  | ColM-R | gagcgactctccgataatgc | 429 | [17] |
| N | ColN-F | agcttggcgagtatcttgga |  |  |
|  | ColN-R | caacacagccccgaataaac | 401 | [17] |
| S4 | ColS4-F | tatatggcccaactgctggt |  |  |
|  | ColS4-R | cgtaaggacggacacctgtt | 456 | [17] |
| U | ColU-F | tgattgctgcgagaaaaatg |  |  |
|  | ColU-R | tctgacagcctctccctgtt | 485 | [17] |
| Y | ColY-F | gcaggcagaaaagaacaagg |  |  |
|  | ColY-R | cggacgttatttgccttcat | 477 | [17] |
| 5 | Col5-F | cattggcaaaagcgaaatct |  |  |
|  | Col5-R | tgcaactctggaaacaatcg | 443 | [17] |
| 10 | Col10-F | ggttaccggatttcctggat |  |  |
|  | Col10-R | ttctagatgcttggcccact | 448 | [17] |
| mB17 | microcin B17-F | tcacgccagtctccattaggtgttggcatt |  |  |
|  | microcin B17-R | ttccgccgctgccaccgtttccaccactac | 135 | [34] |
| mC7 | microcin C7-F | cgttcaactgttgcaatgct |  |  |
|  | microcin C7-R | agttgaggggcgtgtaattg | 134 | [17] |
| mH47 | microcin H47-F | cactttcatcccttcggattg |  |  |
|  | microcin H47-R | agctgaagtcgctggcgcacctcc | 227 | [34] |
| mJ25 | microcin J25-F | tcagccatagaaagatataggtgtaccaat |  |  |
|  | microcin J25-R | tgattaagcattttcattttaataaagtgt | 175 | [34] |
| mL | microcin L-F | ggtaaatgatatatgagagaaataacgtta |  |  |
|  | microcin L-R | tttcgctgagttggaatttcctgctgcatc | 233 | [34] |
| mM | M-DS2009-F | cgtttattagcccgggattt |  |  |
|  | M-DS2009-R | gcagacgaagaggcacttg | 166 | [20] |
| mV | microcin V-F | cacacacaaaacgggagctgtt |  |  |
|  | microcin V-R | cttcccgcagcatagttccat | 680 | [34] |
| *α-hly* | F | aacaaggataagcactgttctggct | 1177 | [25] |
|  | R | accatataagcggtcattcccgtca |  |  |
| *afa* | F | gctgggcagcaaactgataactctc | 750 | [25] |
|  | R | catcaagctgtttgttcgtccgccg |  |  |
| *aer* | F | taccggattgtcatatgcagaccgt | 602 | [25] |
|  | R | aatatcttcctccagtccggagaag |  |  |
| *cnf*1 | F | aagatggagtttcctatgcaggag | 498 | [25] |
|  | R | cattcagagtcctgccctcattatt |  |  |
| *sfa* | F | ctccggagaactgggtgcatcttac | 410 | [25] |
|  | R | cggaggagtaattacaaacctggca |  |  |
| *pap* | F | gacggctgtactgcagggtgtggcg | 328 | [25] |
|  | R | atatcctttctgcagggatgcaata |  |  |
| pCVD432 | F | ctggcgaaagactgtatcat | 630 | [26] |
|  | R | caatgtatagaaatccgctgtt |  |  |
| *ial* | F | ggtatgatgatgatgagtcca | 650 | [27] |
|  | R | ggaggccaacaattatttcc |  |  |
| *lt* | F | ggcgacagattataccgtgc | 450 | [27] |
|  | R | cggtctctatattccctgtt |  |  |
| *st* | F | atttttctttctgtattgtctt | 190 | [27] |
|  | R | cacccggtacaagcaggatt |  |  |
| *bfpA* | F | aatggtgcttgcgcttgctgc | 324 | [27] |
|  | R | gccgctttatccaacctggta |  |  |
| *eaeA* | F | gacccggcacaagcataagc | 384 | [27] |
|  | R | ccacctgcagcaacaagagg |  |  |
| *ipaH* | F | tccgaattccttgaccgccttt | 690 | [28] |
|  | R | ttcgaattcacgcatcacctgtgca |  |  |
| *iucC* | F | gcgaattcggcgatgaccgctactg | 810 | [29] |
|  | R | gcgaattccagcgtgaagccagtg |  |  |
| *fimA* | F | ggcgaattctgttctgtcggctctgtc | 510 | [28] |
|  | R | ttggaattcaaccttgaaggtcgcatc |  |  |
| *stx1* | F | ataaatcgccattcgttgactac | 180 | [30] |
|  | R | agaacgcccactgagatcatc |  |  |
| *stx2* | F | ggcactgtctgaaactgctcc | 255 | [30] |
|  | R | tcgccagttatctgacattctg |  |  |
| *ehly* | F | gcatcatcaagcgtacgttcc | 534 | [30] |
|  | R | aatgagccaagctggttaagct |  |  |
| *fimH* | F | catgccatggccatgaaacgagttattacc | 903 | [31] |
|  | R | cccaagcttttgataaacaaaagtcac |  |  |
